# Unraveling LncRNA GAS5 in Atherosclerosis: Mechanistic Insights and Clinical Translation

**DOI:** 10.3390/biology14060697

**Published:** 2025-06-13

**Authors:** Yu Wei, Quanye Luo, Xiang Li, Xi Liu, Zheyu Yang, Qinhui Tuo, Wen Chen

**Affiliations:** 1 Key Laboratory of Vascular Biology and Translational Medicine, Medical School, Hunan University of Chinese Medicine, Changsha 410208, China; 20223776@stu.hnucm.edu.cn (Y.W.); 20223777@stu.hnucm.edu.cn (Q.L.); lx2254344592@stu.hnucm.edu.cn (X.L.); 202108010111@stu.hnucm.edu.cn (X.L.); 202208010543@stu.hnucm.edu.cn (Z.Y.); 2 Key Laboratory for Quality Evaluation of Bulk Herbs of Hunan Province, School of Pharmacy, Hunan University of Chinese Medicine, Changsha 410208, China; 3 Basic Research Center of Integrated Chinese and Western Medicine on Prevention and Treatment of Vascular Diseases, Medical School, Hunan University of Chinese Medicine, Changsha 410208, China

**Keywords:** lncRNA, *GAS5*, atherosclerosis, cardiovascular disease

## Abstract

Atherosclerosis, a chronic inflammatory disease driving cardiovascular events, involves complex molecular networks where long non-coding RNAs (lncRNAs) are key regulators. This review synthesizes current knowledge on lncRNA Growth Arrest-Specific 5 *(GAS5)* in atherosclerosis, covering its expression, multifaceted roles in vascular cells, and molecular mechanisms. *GAS5* is significantly upregulated in atherosclerotic plaques, exerting complex, cell-specific effects on vascular smooth muscle cells, macrophages, and endothelial cells. *GAS5* modulates crucial pathophysiological processes like cell proliferation, apoptosis, inflammation, lipid metabolism, and foam cell formation, primarily by acting as a competing endogenous RNA (ceRNA) and through direct protein interactions. While promising as a biomarker, circulating *GAS5* levels require further validation. Therapeutic strategies targeting *GAS5*, including antisense oligonucleotides (ASO) and small-molecule compounds, are under investigation. In conclusion, *lncRNA GAS5* is a critical regulatory node in atherosclerosis pathobiology, offering significant opportunities for novel diagnostic and therapeutic interventions. Further research is vital to elucidate its intricate roles and translate these findings into clinical applications for atherosclerotic cardiovascular disease.

## Simple Summary

Hardening of the arteries, a widespread health issue called atherosclerosis, often leads to serious heart problems. This paper reviews what scientists have learned about a tiny part of our cells called *GAS5* and how it is involved in this artery disease. The main goal of this review is to summarize the current understanding of *GAS5*’s complex role in blood vessels during atherosclerosis, which could highlight new paths for treatment. The reviewed research indicates that *GAS5* is often found in higher amounts in unhealthy arteries and influences important processes like swelling (inflammation), the behavior of cells in blood vessels, and the buildup of fatty blockages. However, its exact effects can vary depending on the specific cell type and situation. By bringing together these findings, this review concludes that *GAS5* is a key player in how artery hardening begins and worsens. This knowledge could help doctors better detect or understand the disease and guide future research toward new ways to prevent or treat atherosclerosis, offering significant benefits for public health by tackling heart disease.

## 1. Introduction

### 1.1. The Significance of Atherosclerosis

Atherosclerosis, a significant global health issue, is the fundamental pathological driver behind most cardiovascular diseases, including myocardial infarction and ischemic stroke [1]. The epidemiological survey revealed that, as of 2020, the distribution of atherosclerotic lesions among individuals aged 30–79 years was characterized as follows: carotid intima-media thickening demonstrated a prevalence of 27.6% (95% CI 16.9–41.3), affecting approximately 1 billion individuals; carotid plaque formation exhibited a prevalence of 21.1% (95% CI 13.2–31.5), impacting around 800 million individuals; and carotid stenosis presented a prevalence of 1.5% (95% CI 1.1–2.1), involving 57.79 million individuals [2]. These findings underscore the critical need for enhanced prevention and control strategies against atherosclerotic cardiovascular diseases.

Atherosclerosis manifests as a chronic vascular pathology originating from endothelial dysfunction, where compromised vascular integrity permits progressive lipid deposition within the arterial intima. This pathological cascade drives the progressive accumulation of foam cells and atheromatous streaks, culminating in plaque development accompanied by vascular lumen narrowing. Plaque rupture can result in severe complications such as thrombosis, ischemia, tissue infarction, and ischemic stroke [3]. In advanced plaques, the release of inflammatory factors and macrophage apoptosis further exacerbate lesion progression by fostering necrotic core formation [4]. The pathogenesis of atherosclerosis involves intricate crosstalk among diverse cellular components and signaling pathways, encompassing macrophage polarization, foam cell formation, phenotypic switching of vascular smooth muscle cells (VSMCs), and endothelial dysfunction, collectively highlighting the multifaceted nature of this disease process [5].

Current research on atherosclerosis highlights the persistent need to elucidate its pathological mechanisms and refine therapeutic strategies. The ongoing discovery of novel regulatory molecules and therapeutic targets remains crucial for developing effective preventive and clinical interventions against this complex vascular disorder. Of particular interest are lncRNAs, a class of epigenomic regulators increasingly recognized for their pivotal roles in modulating atherosclerotic progression through gene expression regulation and cellular functional modulation.

### 1.2. Contribution of lncRNAs to Atherosclerosis

The vast majority of the mammalian genome, once considered transcriptional “noise”, is now known to generate, via transcription, what are termed non-protein-coding RNAs (ncRNAs) [6]. Notable among this group are lncRNA, which form an extensive class exhibiting diverse functions, defined as transcripts exceeding 200 nucleotides in length that lack significant open reading frames (ORFs) or protein-coding potential, with functional diversity spanning multiple biological systems [7,8,9].

LncRNAs modulate diverse cellular mechanisms—including genomic transcription, translational regulation, along with proliferative activity, programmed death, metastatic potential, and motility—thereby driving disease pathogenesis [10,11,12,13,14,15]. They can serve as scaffolds or decoys to regulate gene expression at specific loci, recruit chromatin-modifying enzymes, or bind to protein complexes to modulate protein modifications [16]. Notably, certain lncRNAs operate through ceRNA mechanisms, forming dynamic interactions with 20–25 nt non-coding RNAs (microRNAs) to modulate transcriptional outputs [17,18]. Atherosclerotic pathogenesis and its complications are substantially governed by their regulatory dominance [19].

**Figure 1:**
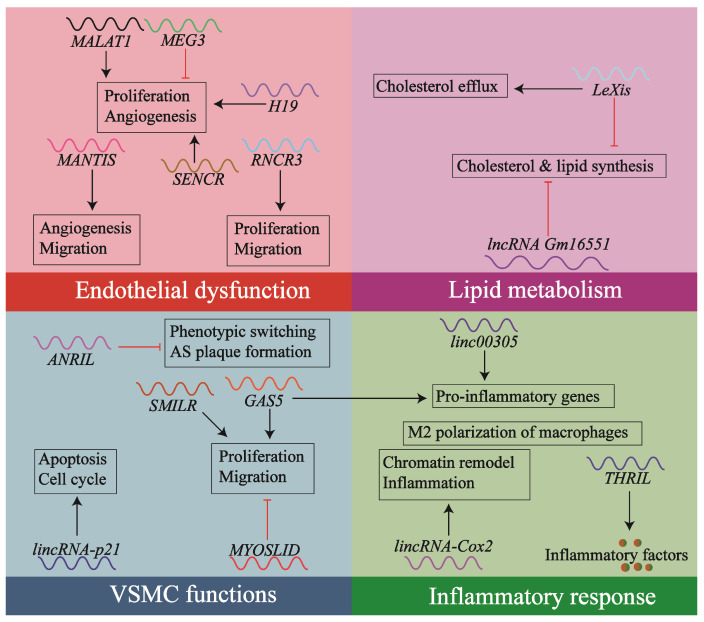
Regulating lncRNAs-related processes in atherosclerosis. This schematic diagram contains several lncRNAs that play a role in atherosclerosis by regulating atherosclerosis-related cellular functions and pathological processes, including endothelial cell dysfunction, VSMC proliferation, inflammatory response, and lipid metabolism disorders. Arrows and T-bars in the diagram represent promotion/activation and inhibition/suppression.

Clinically, lncRNA clusters demonstrate therapeutic potential, particularly in modulating myocardial pathologies. Certain lncRNAs regulate endothelial cells (ECs) proliferation and angiogenesis (Figure 1). For instance, lncRNA *H19* binds to acid phosphatase 5 (ACP5) in ECs, post-transcriptionally modulating ACP5 expression. This process further regulates arterial ECs proliferation and apoptosis, ultimately contributing to atherosclerosis and ischemic stroke [20]. In contrast, other lncRNAs influence VSMCs transdifferentiation processes or vascular remodeling. For example, metformin stimulation upregulates lncRNA *ANRIL* in VSMCs, enabling its interaction with the adenosine 5′-monophosphate (AMP)-activated protein kinase (AMPK) γ subunit. This interaction activates the AMPK pathway, thereby inhibiting VSMC phenotypic switching and atherosclerotic plaque formation [21].

Numerous lncRNAs can regulate atherosclerosis-related processes. For instance, lncRNA *MALAT1*, *MEG3*, *MANTIS*, and *RNCR3* modulate endothelial cell dysfunction; *lincRNA-p21*, *SENCR*, *GAS5*, *MYOSLID*, *SMILR*, and *ANRIL* regulate vascular smooth muscle cell function; *linc00305*, *Cox2*, and *THRIL* influence inflammatory responses; while *Gm16551* and *LeXis* control lipid metabolism. It can be stated that lncRNAs still exhibit substantial potential in atherosclerosis research [22].

### 1.3. The lncRNA GAS5: Involvement in Atherosclerosis

Initially noted for its involvement in regulating cancer cells cycle arrest and programmed cell death, the lncRNA *GAS5* has been suggested to function as a competitive microRNA sponge or protein interaction partner [6]. Emerging evidence indicates that *GAS5* exhibits pleiotropic roles across diverse biological contexts and disease pathologies, including carcinogenesis, autoimmune disorders, metabolic syndromes, and notably, cardiovascular diseases [23,24,25,26,27,28].

Mechanistically, *GAS5* modulates atherosclerosis-relevant cellular processes—such as endothelial dysfunction, macrophage polarization, foam cell formation, phenotypic switching of VSMCs, and lipid metabolism—through context-dependent regulatory effects [29,30,31,32,33,34]. Its expression dynamics and functional outputs demonstrate spatiotemporal heterogeneity contingent upon cell type specificity, disease progression stages, and plaque-specific microenvironmental niches.

This paper examines the diverse roles and intricate contributions of *GAS5* in atherogenesis. We summarize current mechanistic insights into its regulatory pathways, cellular functions, and molecular interactomes within atherosclerotic lesions. Furthermore, we critically evaluate its clinical translational potential, emphasizing its dual candidacy as a disease-specific biomarker and a microenvironment-responsive therapeutic target.

## 2. The Diverse Functions of lncRNA *GAS5*

### 2.1. Genomic and Molecular Characteristics of lncRNA GAS5

The lncRNA *GAS5* was first identified in murine NIH 3T3 cells through subtractive hybridization. Its nomenclature reflects the significant accumulation of its expression levels in cells undergoing growth arrest. Such growth arrest can be triggered by various conditions, including serum starvation, inhibition of protein translation, or cell–cell contact inhibition. These stress-induced growth arrest conditions lead to diminished translation and degradation of RNAs transcribed from genes containing a 5′-terminal oligopyrimidine (5′TOP) motif. Consequently, this reduction results in a marked increase in mature, spliced *GAS5* RNA [35].

In humans, the *GAS5* gene is located on chromosome 1q25.1. *GAS5* serves as a host gene for small nucleolar RNAs (snoRNAs), with its intronic sequences encoding as many as 10 C/D-box snoRNAs. These intronic snoRNAs are implicated in the biogenesis of ribosomal RNA [36,37]. Furthermore, one study in human colorectal cancer (CRC) cell lines demonstrated that *SNORD80*, a snoRNA encoded by a *GAS5* intron, can direct the 2′-O-methylation of *GAS5*. This modification stabilizes *GAS5* and modulates the cellular stress response, thereby establishing a positive feedback regulatory loop [38].

The human *GAS5* gene comprises up to 12 exons and generates multiple splicing variants, which contributes to the diversity of its regulatory functions. Additionally, *GAS5* harbors a small, poorly conserved putative ORF. The spliced ORF of human *GAS5* is relatively limited in size, featuring a premature termination codon within an early exon [39]. This characteristic renders these transcripts susceptible to degradation through the nonsense-mediated mRNA decay (NMD) pathway during translation [40].

### 2.2. Multifaceted Regulation of lncRNA GAS5

**Figure 2:**
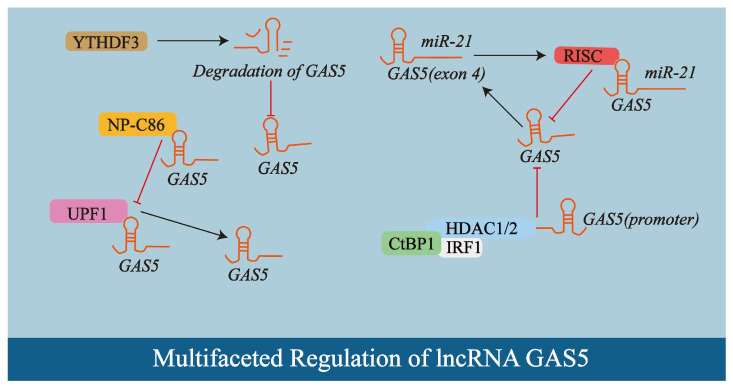
The expression of lncRNA *GAS5* is regulated through multiple mechanisms. This schematic illustrates key regulatory factors: *miR-21* binds to exon 4 of *GAS5*, recruits the RNA-induced silencing complex (RISC) to induce its degradation, establishing a negative feedback loop; the CtBP1-HDAC1/2-IRF1 complex represses *GAS5* transcription by interacting with its promoter; the m6A reader protein YTHDF3 mediates *GAS5* decay; and the small molecule np-C86 stabilizes *GAS5* by specifically binding to it and blocking UPF1-mediated degradation. Arrows and T-bars in the diagram represent promotion/activation and inhibition/suppression. For further details, refer to Section 2.2.

The synthesis and functional roles of the lncRNA *GAS5* are governed by a multitude of factors (Figure 2). Its expression is notably influenced by cellular state, with *GAS5* accumulating in growth-arrested cells, a condition inducible by serum starvation, protein translation inhibitors, or cell–cell contact inhibition [35]. Dysregulation of *GAS5* expression is frequently observed in various pathologies; for instance, it is often downregulated in many cancer types [39] and exhibits markedly lower serum levels in individuals with diabetes [41]. Studies in breast cancer cell lines and murine models have revealed that microRNA-mediated regulation also plays a significant role, as exemplified by *miR-21*, which directly binds to exon 4 of *GAS5*, leading to recruitment of the RNA-induced silencing complex (RISC) and subsequent post-transcriptional downregulation of *GAS5*, forming a mutually negative regulatory feedback loop [42].

Furthermore, studies in human osteosarcoma cells demonstrate that transcription factors and complexes modulate *GAS5* expression; the C-terminal binding protein 1 (CtBP1)-histone deacetylase 1/2 (HDAC1/2)-Interferon regulatory factor 1 (IRF1) complex, for example, can interact with the *GAS5* promoter to repress its expression [43]. RNA-binding proteins and post-transcriptional modifications also contribute to *GAS5* regulation, with experimental systems employing CRC cell lines and murine models demonstrating that the m6A reader protein YTH-domain family member 3 (YTHDF3) negatively regulates *GAS5* by inducing its decay [44], and studies using Lewis lung cancer Luciferase (LLC-Luc) cells and mouse models revealing that the Up-frameshift protein 1 (UPF1)-mediated NMD pathway participates in its modulation [41].

Additionally, small molecules can influence *GAS5* stability and expression; np-C86, for instance, associates with *GAS5* with high specificity in adipose-derived stem cells, preventing UPF1’s interaction with *GAS5*, thereby limiting its degradation and promoting its stabilization and upregulation [40,41]. Given these pleiotropic effects and complex regulatory inputs, *GAS5* likely plays divergent roles in promoting or inhibiting disease processes, such as in atherosclerosis where its function may vary depending on specific cellular and microenvironmental contexts, underscoring the necessity for further in-depth investigation.

### 2.3. Diverse Functional Mechanisms of lncRNA GAS5

**Figure 3:**
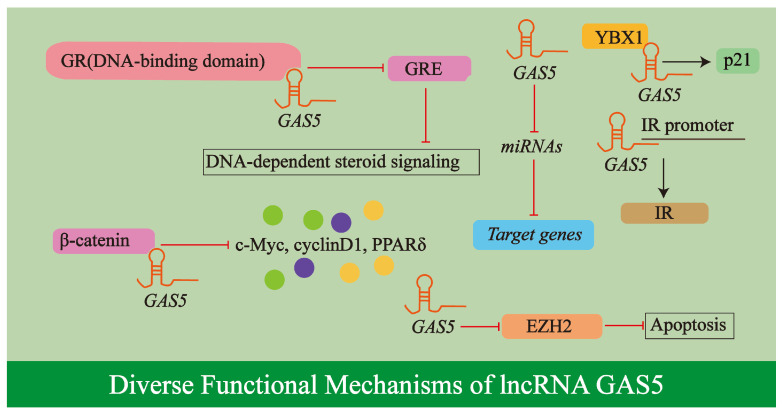
Diverse functional mechanisms of lncRNA *GAS5*. This schematic illustrates the molecular mechanisms of lncRNA *GAS5*, encompassing glucocorticoid receptor (GR) inhibition through competitive binding to the glucocorticoid response element (GRE); its role as a competing endogenous RNA (ceRNA) that sponges miRNAs (e.g., *miR-21*, *miR-96-5p*, and *miR-28a-5p*); protein interaction-mediated regulation via β-catenin and YBX1; and direct modulation of gene expression by targeting promoters such as INSR or repressing EZH2 transcription. Arrows and T-bars in the diagram represent promotion/activation and inhibition/suppression. For further details, refer to Section 2.3.

The lncRNA *GAS5* executes its biological roles through a variety of molecular mechanisms (Figure 3). As a growth arrest and starvation-linked inhibitor, *GAS5* affects the Glucocorticoid Receptor (GR) [35], as demonstrated in HeLa cells, functioning as a riborepressor by acting as a decoy for the glucocorticoid response element (GRE) through specific interaction with the GR’s DNA-binding domain, thereby inhibiting DNA-dependent steroid signaling [40], shown in adipose-derived stem cells from type 2 diabetes patients.

As a versatile ceRNA, *GAS5* binds multiple miRNAs such as *miR-21* [45], *miR-96-5p* [46], and *miR-28a-5p* [47], effectively neutralizing their ability to repress target genes [36]. This sponge-like activity enables *GAS5* to broadly regulate downstream signaling networks.

Furthermore, *GAS5* engages in protein interactions that can modify protein activity, structure, and localization; for instance, it interacts and colocalizes with β-catenin to regulate the cellular functions of ECs and VSMCs via the β-catenin signaling pathway [48] and directly binds Y-box binding protein 1 (YBX1) to form a self-reinforcing regulatory circuit that controls the expression of the downstream target gene p21 [45]. Beyond these interactions, *GAS5* directly modulates the activity of subsequent genetic targets, such as binding to the insulin receptor (IR) promoter to regulate its expression and promoting apoptosis in bladder cancer cells by downregulating the transcription of enhancer of zeste homolog 2 (EZH2) [40,49].

The structural integrity of *GAS5*, characterized by a modular secondary structure, allows its distinct modules to function independently under various cellular stress conditions, each associated with different cell survival regulatory functions [37]. Collectively, these diverse mechanisms—GR inhibition, ceRNA activity, protein interactions, and regulation of gene expression—are orchestrated within complex regulatory networks, forming the basis for *GAS5*’s cell-specific and sometimes contradictory effects observed in different pathologies, including atherosclerosis.

### 2.4. lncRNA GAS5 in Human Diseases: A Broad Spectrum

**Figure 4:**
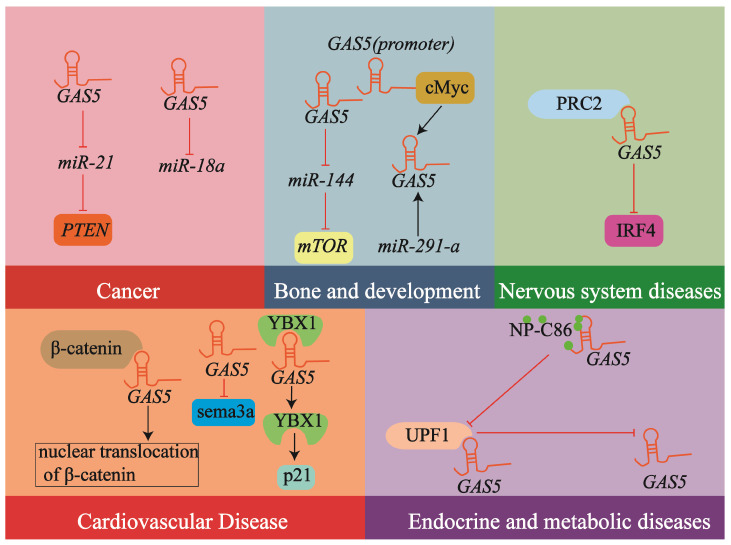
Interaction molecules and signaling pathways of lncRNA *GAS5* in different diseases. This schematic diagram contains some of the regulatory pathways and mechanisms of *GAS5* mentioned in different disease domains. Arrows and T-bars in the diagram represent promotion/activation and inhibition/suppression.

*GAS5* is significantly involved in a multitude of biological pathways and diseases (Table 1, Figure 4).

Cancer: *GAS5* is broadly regarded as a tumor suppressor, with its down-regulation observed across multiple cancer types and often associated with unfavorable prognosis, and it exerts anti-cancer effects by regulating the *miR-21*/*PTEN* axis [42,50]. *GAS5* is involved in regulating the survival, migration, invasion, cell cycle progression, and proliferation of human prostate cancer cells, and it exerts these effects by modulating *miR-18a*. It is also associated with chemoresistance in tumors [51].

Cardiovascular disease: *GAS5* plays a significant role in the cardiovascular system, participating in hypertension-induced vascular remodeling, cardiomyocyte apoptosis, and the regulation of apoptosis and proliferation in VSMCs by modulating β-catenin signaling (associated with vascular remodeling) [48], sema3a (linked to cardiomyocyte apoptosis) [52], and interacting with YBX1 and *miR-21* (related to VSMC apoptosis) [45].

Endocrine and metabolic diseases: *GAS5* is a significant factor concerning these conditions. *GAS5* levels are markedly lower in serum samples from individuals diagnosed with type 2 diabetes mellitus (T2DM) and it affects insulin signaling and glucose uptake by binding to UPF1 and regulating insulin receptor expression [40].

Nervous system diseases: As the principal immune effectors within the central nervous system, microglia can polarize into different functional states, with the M1 state generally associated with pro-inflammation, while the M2 state is the opposite. Research indicates that *GAS5* plays a role in nervous system diseases, such as multiple sclerosis (MS), by binding to PRC2 and recruiting it to the IRF4 gene promoter, thereby inhibiting microglial M2 polarization and exacerbating demyelination [53].

Bone and development: *GAS5* is a key regulator of chondrocyte growth, differentiation, and development. The expression of *GAS5* negatively regulates the levels of *miR-144*. Given that *miR-144* targets and inhibits mTOR, the suppressive effect of *GAS5* on *miR-144* leads to an increased expression level of mTOR. This ultimately results in the inhibition of autophagy in osteoarthritis chondrocytes [54]. *GAS5* also plays a critical role in embryonic development and stem cell biology as a key modulator of pluripotency and self-renewal in mouse and human embryonic stem cells, where its expression is positively regulated by *miR-291-a* and the transcription factor cMyc (via direct binding to the *GAS5* promoter), forming a regulatory network that governs self-renewal and pluripotency in mouse embryonic stem cells (mESCs) [55].

### 2.5. The Potential of lncRNA GAS5 in Atherosclerosis

As mentioned above, *GAS5* can widely regulate the progression of different diseases, including cardiovascular diseases, through various mechanisms. To illustrate, elevated concentrations of *GAS5* are detected in rat models of atherosclerosis. In experimental studies using apolipoprotein E-deficient (ApoE^−/−^), mice demonstrated that high-fat diet feeding induces atherosclerosis development, with subsequent analysis revealing significantly elevated *GAS5* expression levels in blood samples from atherosclerotic mice compared to control groups [56]. This observed upregulation of *GAS5* suggests its potential involvement in regulating atherosclerotic progression.

Exosomal *GAS5* is additionally capable of modulating programmed cell death in macrophages and endothelial cells pertinent to atherosclerosis. Studies have demonstrated that in oxidized low-density lipoprotein (ox-LDL)-stimulated THP-1 macrophages, overexpression of *GAS5* significantly enhances cellular apoptosis by upregulating pro-apoptotic factors (Caspase 3/7/9 and P53). Conversely, *GAS5* knockdown suppresses apoptosis. Notably, exosomes secreted by ox-LDL-treated THP-1 macrophages exhibit enriched *GAS5* content. These exosomes can be internalized by vascular endothelial cells, leading to a marked increase in endothelial cell apoptosis accompanied by upregulated protein expression of Caspase 3/7/9 and P53 [56,57]. Nevertheless, the precise roles and operational pathways of *GAS5* concerning atherosclerosis remain to be thoroughly clarified.

Given the known role of *GAS5* in apoptosis, inflammation, and lipid metabolism disorders, as well as its upregulated expression in atherosclerosis models, it is reasonable to further elucidate how *GAS5* is involved and the specific mechanisms driving its effects during the advancement of atherosclerosis [30,56]. Consequently, *GAS5* could emerge as a promising focus for developing atherosclerosis treatments. Previous studies which use THP-1 cells have identified that *GAS5* contains putative binding sites for *miR-135a*, as confirmed by dual-luciferase reporter assays. Subsequent experimental evidence demonstrates that *GAS5* functions as a molecular sponge for *miR-135a*, with its silencing shown to mitigate atherosclerotic progression through *miR-135a* upregulation [56].

## 3. Expression and Impact of lncRNA *GAS5* in Atherosclerosis

### 3.1. Expression in Atherosclerotic Plaques

Multiple studies consistently indicate that lncRNA *GAS5* is significantly upregulated in atherosclerotic plaques compared to normal vascular tissue. The samples for these studies originated from human carotid or aortic plaques [58], as well as various atherosclerosis animal models such as ApoE^−/−^ mice and atherosclerosis rat models [59,60]. This high-specific expression in lesion sites suggests that *GAS5* may play a crucial role in the development and progression of atherosclerosis.

### 3.2. Circulating lncRNA GAS5 Levels and Their Clinical Relevance

In addition to changes in *GAS5* expression within plaques tissue, researchers have also focused on the levels of *GAS5* in circulating blood and its potential as a biomarker. However, existing study results are contradictory.

Studies on circulating lncRNA *GAS5* levels have yielded conflicting results depending on the cardiovascular context. Some reports indicate that plasma *GAS5* is significantly elevated in patients with coronary heart disease (CHD) or acute ischemic stroke (AIS) compared to healthy controls. In CHD patients, median *GAS5* expression was more than double that of controls (2.270 vs. 0.999), showing exceptional diagnostic efficacy (AUC = 0.915) and a positive correlation with disease severity and inflammatory markers. These elevated levels may also predict the risk of recurrent AIS [26]. Similarly, in AIS cases, *GAS5* expression was markedly higher, with a diagnostic cutoff of 1.865 providing 93.3% specificity [26,61].

In contrast, other research links reduced *GAS5* expression to different pathologies. In hypertensive populations, lower *GAS5* is associated with an increased risk of asymptomatic organ damage (AOD), with levels in affected individuals dropping to a median fold change of 0.25. This downregulation demonstrated a high capacity for detecting both hypertension and AOD [62]. Furthermore, other studies have found significantly lower plasma *GAS5* in patients with coronary artery disease (CAD) [63], suggesting its downregulation could serve as a relatively specific biomarker for the condition [27].

The observed variations in circulating *GAS5* levels across different studies may arise from differences in study design, including heterogeneity in patient populations (such as various subtypes of cardiovascular disease and the presence of comorbidities like diabetes), sample types (plasma vs. serum), insufficient standardization of detection methods, and the inherently complex dynamic regulation of lncRNA release into circulation [58]. These conflicting results suggest that larger confirmatory studies in well-defined patient cohorts are necessary before circulating *GAS5* can be considered a reliable biomarker for atherosclerosis.

### 3.3. Association with Clinical Parameters

Overall, *GAS5* expression levels correlate with atherosclerosis and its related clinical indicators, both in plaque tissue and circulating blood. *GAS5* expression in plaques is positively correlated with macrophage activation [64]. Circulating *GAS5* levels are positively associated with the degree of stenosis in the coronary arteries (Gensini score) and acute ischemic stroke (NIHSS score) [26,61]. Circulating *GAS5* levels are positively correlated with inflammatory markers (CRP, TNF-α, IL-6, IL-17A) and negatively correlated with HDL-C [26]. Circulating *GAS5* levels can predict the risk of AIS recurrence [61]. *GAS5* expression levels are associated with AOD in hypertensive patients [62]. A single nucleotide polymorphism (SNP), rs145204276, in the *GAS5* promoter region is related to the risk of atherosclerosis [65,66].

## 4. Cell-Specific Mechanisms of *GAS5* in Atherosclerosis

### 4.1. lncRNA GAS5 and VSMCs

#### 4.1.1. Regulation of VSMCs Proliferation and Apoptosis

The p53–p300 pathway: lncRNA *GAS5* enhances p53 protein stability and inhibits its ubiquitination and degradation by directly binding to the tumor suppressor p53 and its coactivator p300, forming a ternary complex. This activation leads to increased expression of subsequent gene targets, including p21 and pro-apoptotic factor PMA-induced protein 1 (NOXA), inducing G1 phase cell cycle arrest and mitochondria-dependent apoptosis in VSMCs. In pathological conditions like vascular injury, downregulation of *GAS5* results in loss of p53 activity, promoting abnormal proliferation and neointima formation of VSMCs. Conversely, adenovirus-mediated overexpression of *GAS5* can significantly inhibit intimal hyperplasia after rat carotid balloon injury by restoring p53 function [67].

The *miR-21*/programmed cell death protein 4 (*PDCD4*) axis: lncRNA *GAS5* upregulates PDCD4 protein expression by directly binding to *miR-21*, thereby relieving *miR-21*’s post-transcriptional inhibition of its target gene PDCD4. As a pro-apoptotic and anti-proliferative factor, PDCD4 can counter the aberrant VSMC proliferative and migratory activities that the platelet-derived growth factor BB (PDGF-BB) promotes. Consequently, the *GAS5*/*miR-21*/*PDCD4* axis may slow down the formation of atherosclerotic plaques by inhibiting excessive proliferation and migration of VSMCs [68].

The *miR-145-5p* pathway: The expression of *GAS5* is significantly upregulated in the aortic tissue of mice induced by di(2-ethylhexyl) phthalate (DEHP). However, further cellular experiments reveal that DEHP stimulation can induce VSMCs proliferation and promote apoptosis, during which the expression of *GAS5* increases and it binds to *miR-145-5p* to downregulate its expression. This finding contradicts other studies, suggesting that the role of *GAS5* is largely influenced by tissue type, cell type, and the context of drug stimulation [30]. This suggests that *GAS5* may have a regulatory pattern different from the general one under specific stimuli (such as DEHP).

The ATPase/L31 axis: In one study, rats administered benzo[a]pyrene (BaP)—a pro-oxidant present in tobacco smoke that accelerates atherosclerosis and induces a synthetic VSMC phenotype—exhibited elevated levels of lncRNA *GAS5* and mitochondrial ATPase, along with reduced ribosomal protein L31 in isolated VSMCs [29]. These findings indicate that lncRNA *GAS5* influences VSMC proliferation and phenotypic transformation by modulating mitochondrial and ribosomal activity.

#### 4.1.2. Regulation of VSMCs Migration

The *miR-21* pathway: Targeting *miR-21*, *GAS5* suppresses PDGF-BB-induced VSMCs migration by sequestering the miRNA and derepressing its targets. However, overexpression of *miR-21* abolishes *GAS5*’s inhibitory effect, demonstrating a dose-dependent antagonism in VSMCs motility regulation [68]. *miR-21* is a regulatory factor of the protein kinase B (Akt)/extracellular signal-regulated kinases (ERK) signaling pathway, and *GAS5* may also influence the proliferation and migration of VSMCs through the *miR-21*/*Akt*/*ERK* signaling pathway [69].

*miR-23b-3p*/*KCNK3* axis: In hypoxic rodent models and cultured pulmonary artery smooth muscle cells (PASMCs), *GAS5* downregulation correlates with increased proliferation and migration. This phenotype arises from impaired *miR-23b-3p* sponging by *GAS5*, which disrupts KCNK3 signaling and drives pathological PASMC remodeling [70].

#### 4.1.3. Phenotypic Switching of VSMCs

The phenotypic switching of VSMCs represents a hallmark pathological alteration in atherosclerosis, with emerging evidence suggesting the lncRNA *GAS5* may regulate this process.

Studies have shown that *GAS5* suppresses *miR-665* to upregulate Syndecan-1 (SDC1) expression, thereby exerting anti-senescence effects. SDC1 modulates the phenotypic switching of VSMCs, and knockdown of SDC1 has been demonstrated to promote the transition toward a synthetic phenotype with enhanced proliferative activity, which is accompanied by downregulation of the contractile marker α-SMA. These findings suggest that *GAS5* may inhibit synthetic phenotype transition through SDC1 upregulation [71,72,73].

**Figure 5:**
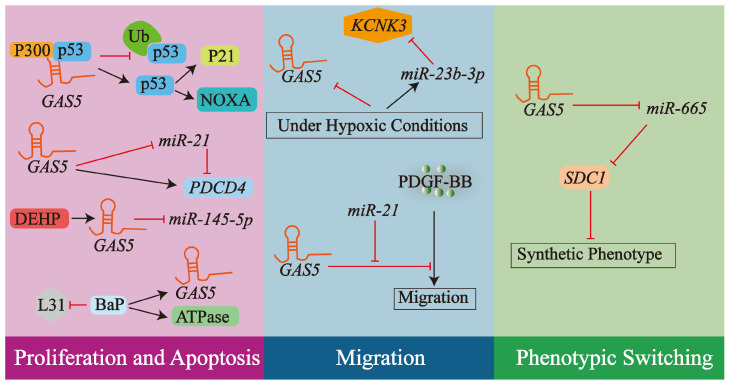
LncRNA *GAS5* orchestrates multifaceted responses in VSMCs impacting atherosclerosis. This schematic depicts how *lncRNA GAS5* influences VSMC proliferation, apoptosis, migration, and phenotypic switching. Its regulatory effects are mediated through direct interactions with protein signaling pathways, such as the p53–p300 axis, and by acting as a ceRNA for various miRNAs, thereby modulating downstream gene expression critical for VSMC function. Arrows and T-bars in the diagram represent promotion/activation and inhibition/suppression. For further details, refer to Section 4.1.

Collectively, these findings underscore the complex and multifaceted regulatory roles of lncRNA *GAS5* in VSMC biology, influencing proliferation, apoptosis, migration, and phenotypic state through diverse molecular pathways. The principal mechanisms by which lncRNA *GAS5* exerts these effects on VSMCs are summarized in Figure 5.

### 4.2. lncRNA GAS5 in Macrophages

#### 4.2.1. Modulation of Macrophage Inflammatory Responses

The *miR-221*/monocyte chemotactic protein 1 *(MCP-1*) axis: *GAS5* exacerbates macrophage inflammation by directly inhibiting *miR-221*. Elevated *GAS5* levels intensify the release of pro-inflammatory cytokines (IL-6, IL-1β, TNF-α) and the chemokine MCP-1 from macrophages stimulated by ox-LDL. In contrast, diminishing *GAS5* activity can nullify these outcomes. Significantly, an increase in *miR-221* abundance can counteract the enhanced inflammatory response driven by *GAS5* [74].

The *miR-135a* pathway: *GAS5* is capable of direct interaction with *miR-135a*, leading to the inhibition of its expression. Additionally, *GAS5* serves as a molecular sponge that sequesters *miR-135a*, thereby promoting inflammation (levels of IL-1β, IL-6, TNF-α) [56]. Knockdown of *GAS5* alleviates inflammation by upregulating *miR-135a*. Knockdown of *miR-135a* partially reverses the inhibition of inflammatory cytokine release induced by si-*GAS5*. Silencing *GAS5* significantly alleviated pro-inflammatory activation and oxidative damage in modified lipoprotein-exposed macrophages [75].

By upregulating Annexin A2 (ANXA2): *GAS5* upregulates the expression of ANXA2. In vitro experiments indicate that both *GAS5* and ANXA2 promote macrophage inflammation (IL-1β and TNF-α levels). ANXA2 exhibits dual cytoplasmic and plasmalemmal localization across multiple lineages, where it inhibits PCSK9-mediated intracellular cholesterol accumulation. In macrophages, lncRNA *GAS5* increases ANXA2 protein and promotes inflammatory processes [64].

#### 4.2.2. Regulation of Macrophage MMPs

Mechanistic investigations revealed that lncRNA *GAS5* modulates ox-LDL-mediated inflammatory signaling through miRNA sponging. Specifically, *lncRNA GAS5* competitively binds *miR-221* (a negative regulator of macrophage activation), thereby attenuating its suppression on inflammatory cytokine production and metalloproteinase biosynthesis [74]. This miRNA sequestration effectively relieves the brake on pro-atherogenic pathways, exacerbating extracellular matrix degradation via MMP-2/9 overactivation and compromising plaque structural integrity [74,76].

#### 4.2.3. Lipid Metabolism Disorders

Lipid metabolism disorders: Additional research in murine atherosclerosis models and ox-LDL-treated macrophages has revealed that knocking down lncRNA *GAS5* boosts *miR-135a*, thereby attenuating atherogenic lipoprotein fractions and pro-inflammatory mediators (IL-1β, IL-6, TNF-α), while elevating high-density lipoprotein cholesterol (HDL-C). This effect entails downregulation of peroxisome proliferator-activated receptor α (PPARα) and upregulation of carnitine palmitoyltransferase 1 (CPT1), indicating that lncRNA *GAS5* knockdown curtails both lipid metabolic disorders and inflammation [56].

Lipid accumulation: Mechanistic studies reveal lncRNA *GAS5* critically regulates macrophage lipid accumulation, the pivotal cellular transformation driving atherosclerotic progression. Subcellular localization analyses confirm nuclear enrichment of lncRNA *GAS5* is highly expressed in THP-1-derived foam cells. Elevated lncRNA *GAS5* augments lipid accumulation in macrophages and directly binds EZH2, leading to impaired outward transport of cholesterol alongside lower levels of ATP-binding cassette transporter A1 (ABCA1) [32]. Notably, ABCA1 and ABCG1 govern macrophage cholesterol efflux, promoting the generation of apolipoprotein A-I (ApoA-I) and preventing atherosclerotic lesions [33]. By disrupting ABCA1, lncRNA *GAS5* reduces cholesterol efflux and drives foam cell generation [32,33].

#### 4.2.4. Macrophage Apoptosis and Proliferation

*miR-128-3p*/fibulin-2 (*FBLN2*) axis: lncRNA *GAS5* plays a role in regulating macrophage proliferation and apoptosis. In ox-LDL-treated THP-1 cells, lncRNA *GAS5* expression is upregulated while *miR-128-3p* is downregulated. Elevated *GAS5* upregulates FBLN2 via *miR-128-3p* sponging. Overexpression of lncRNA *GAS5* leads to an increase in FBLN2 levels. In contrast, lncRNA *GAS5* knockdown results in upregulation of Cyclin D1 and Bcl-2, while downregulating Bax and cleaved caspase-3, ultimately promoting cell proliferation. Thus, lncRNA *GAS5* knockdown can enhance proliferation and inhibit apoptosis in ox-LDL-treated THP-1 cells via the *miR-128-3p*/*FBLN2* pathway [77].

Upregulation of ANXA2: In vitro experiments indicate that *GAS5* promotes macrophage apoptosis by upregulating ANXA2, accompanied by changes in Bax, Bcl-2, and Caspase-3 expression [64].

#### 4.2.5. Regulation of Foam Cell Formation

Studies indicate that DEHP treatment markedly elevates *GAS5* levels within macrophages. Functioning as a ceRNA for *miR-145-5p*, *GAS5* directly binds to *miR-145-5p*, relieving its inhibition on target genes. This action enhances lipid uptake by macrophages, stimulates foam cell development, and concurrently increases plasma total cholesterol (TC), low-density lipoprotein (LDL-C), and triglycerides (TG) [30]. This exacerbates lipid metabolic disorders, potentially leading to the formation of necrotic cores in plaques.

**Figure 6:**
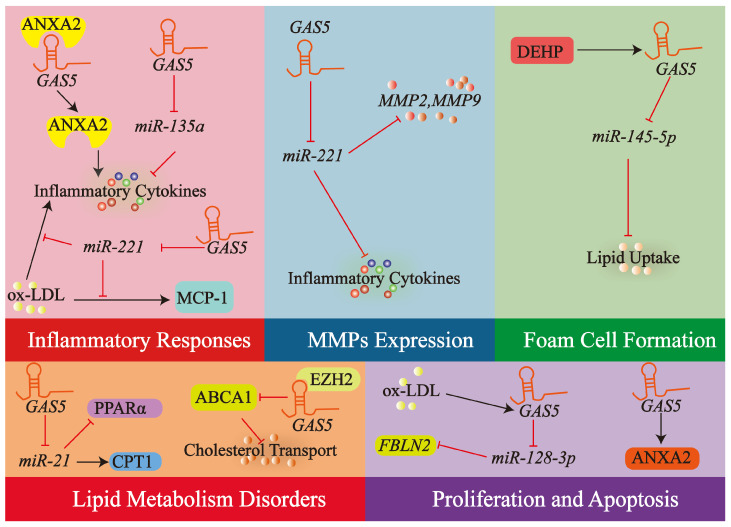
LncRNA *GAS5* modulates key atherogenic functions of macrophages. This illustration summarizes how lncRNA *GAS5* regulates critical macrophage activities in atherosclerosis, including inflammatory responses, MMPs production, lipid metabolism, cholesterol efflux, apoptosis, proliferation, and foam cell formation. These modulatory effects are achieved through *GAS5* acting as a ceRNA for relevant miRNAs and through its interactions with key proteins and signaling cascades involved in macrophage pathobiology. Arrows and T-bars in the diagram represent promotion/activation and inhibition/suppression. Refer to Section 4.2 for detailed mechanisms.

Taken together, lncRNA *GAS5* emerges as a critical modulator of macrophage activity in the context of atherosclerosis, impacting inflammatory responses, MMPs regulation, lipid handling, cell survival, foam cell development. A schematic representation of these diverse regulatory functions of lncRNA *GAS5* in macrophages is provided in Figure 6.

### 4.3. lncRNA GAS5 and Endothelial Cells

#### 4.3.1. Endothelial Cell Proliferation and Apoptosis

The caspase signaling pathway: One study revealed that lncRNA *GAS5* is present in exosomes secreted by ox-LDL-stimulated THP-1 cells [57]. Exosomes, which are small membrane vesicles, facilitate intercellular communication through the transfer of proteins and RNA. Endothelial uptake of lncRNA *GAS5*-transfected THP-1 exosomes significantly augments apoptosis. Conversely, exosomes from lncRNA *GAS5*-knockdown THP-1 cells suppress endothelial apoptosis and reduce the transcription of pro-apoptotic genes such as p53, caspase3, caspase7, and caspase9 [57].

The *miR-21*/*PDCD4* pathway: PDCD4 is a pivotal regulator of cardiovascular diseases, affecting various cellular processes including apoptosis and proliferation [78,79,80]. Research has found that rs145204276 is located in the *GAS5* promoter region, where the deletion allele (DEL) significantly enhances *GAS5* transcriptional activity compared to the insertion allele (INS), leading to increased *GAS5* expression. Population genotyping shows that individuals with the DEL/DEL genotype have a lower risk of developing atherosclerosis. Mechanistically, *GAS5* operates as a ceRNA for *miR-21*, inhibiting its activity and thereby relieving the suppression of the target gene PDCD4 by *miR-21*. PDCD4 then activates the caspase-3 pathway, promoting endothelial cell apoptosis. Under high glucose (HG) stimulation, endothelial cell growth is significantly inhibited while apoptosis is enhanced, an effect more robust in INS/INS-type cells. Notably, lncRNA *GAS5* silencing promotes endothelial proliferation and curtails apoptosis, especially in INS/INS cells [66].

The *miR-194-3p*/thioredoxin-interacting protein (*TXNIP*) pathway: In the ECs of atherosclerotic rats, the expression of *miR-194-3p* is decreased, while the expression of TXNIP is elevated. *miR-194-3p* targets and regulates TXNIP. Overexpression of *miR-194-3p* enhances the proliferation of ECs and inhibits apoptosis in atherosclerosis, and the knockdown of TXNIP can alleviate atherosclerosis. In the context of atherosclerosis, ECs exhibit impaired proliferation and increased apoptosis, which implies that normal ECs have healthy proliferation and lower apoptosis. Therefore, based on the comparison between the atherosclerosis state and the normal state, it can be inferred that in normal cells, *miR-194-3p* levels are likely higher, supporting normal EC proliferation and preventing apoptosis by maintaining appropriate levels of TXNIP (which are not excessively suppressed). lncRNA *GAS5* can bind to *miR-194-3p*, which itself targets TXNIP. In atherosclerotic rat models, lncRNA *GAS5* is upregulated in arterial tissue. Knocking down lncRNA *GAS5* elevates *miR-194-3p*, lowers TXNIP expression, and thereby stimulates endothelial proliferation while limiting apoptosis [60].

The *miR-221*/*Sirt1* pathway: In the plasma of patients with atherosclerosis, in mouse models, and in ox-LDL-treated human aortic endothelial cells (HAECs), the expression of *miR-221* is significantly elevated, whereas SIRT1 expression is decreased in atherosclerosis. It is known that *miR-221* inhibits the proliferation, migration, and tube formation of HAECs, and that *miR-221* targets and negatively regulates the expression of SIRT1 (which is associated with cell survival). Therefore, in normal cells, the *miR-221*/SIRT1 axis likely supports normal HAEC proliferation, migration, and tube formation by maintaining appropriate levels of SIRT1 [81,82]. *GAS5* may also influence endothelial function via the *miR-221*/*Sirt1* pathway. Elevated *miR-221* levels in atherosclerotic mouse carotid arteries, as well as in ox-LDL-challenged human aortic endothelial cells, interfere with cell proliferation and migration by downregulating sirtuin 1 (Sirt1) [81]. Because *GAS5* can directly suppress *miR-221*, the lncRNA *GAS5*/*miR-221*/*Sirt1* axis may modulate endothelial function during atherosclerosis [82].

The *miR-223*/nicotinamide phosphoribosyltransferase (*NAMPT*) pathway: In normal (young) endothelial progenitor cells (EPCs), NAMPT protein levels are relatively high, which is crucial for maintaining normal proliferation and preventing a senescent state. NAMPT overexpression is known to increase EPC proliferation and inhibit cell senescence, and it is vital for the mobilization and angiogenic functions of EPCs in ischemic diseases. The expression of NAMPT is regulated by *miR-223*, which directly binds to the 3′-untranslated region (3′-UTR) of NAMPT, thereby inhibiting its expression. Therefore, it is proposed that in normal EPCs, the *miR-223*/*NAMPT* axis maintains healthy EPC proliferation and suppresses senescence by appropriately modulating NAMPT levels, where *miR-223* likely does not cause excessive suppression of NAMPT [83]. In human EPCs, which replace damaged endothelium, *GAS5* competitively binds *miR-223*, reducing its activity and concurrently enhancing NAMPT expression. Consequently, EPC proliferation is stimulated, whereas senescence is inhibited [83].

The *miR-33a-5p*/*ABCA1* pathway: ABCA1 is a membrane-associated protein that plays a critical role in the apolipoprotein-mediated lipid efflux pathway. The gene for ABCA1 is directly targeted by *miR-33a-5p*. In normal cells, ABCA1 expression is high while *miR-33a-5p* expression is low, a pattern that is reversed in disease states. This suggests that in normal coronary microvascular endothelial cells (CMECs), the *miR-33a-5p*/ABCA1 axis likely promotes normal ABCA1 expression and its function, particularly in lipid efflux, by maintaining appropriately low levels of *miR-33a-5p* that do not cause excessive suppression [84]. In rats, CMECs subjected to homocysteine (HCY) stress, lncRNA *GAS5* levels decrease, *miR-33a-5p* increases, and ABCA1 expression declines. By sponging *miR-33a-5p*, lncRNA *GAS5* upregulation restores ABCA1, ameliorating CMEC proliferation and preventing apoptosis, thus alleviating HCY-induced endothelial damage [84]

#### 4.3.2. Autophagy in Endothelial Cells

The *miR-26a* pathway: *miR-26a* is a highly conserved miRNA that has been shown to play an important role in development, cell differentiation, apoptosis, and growth. In patients with atherosclerosis and in ox-LDL-treated HAECs, the expression of *miR-26a* is decreased. Overexpression of *miR-26a* alleviates the development of atherosclerosis and prevents endothelial cell apoptosis. Data indicates that under conditions of impaired autophagy and increased apoptosis, such as in atherosclerosis or with ox-LDL treatment, *miR-26a* is downregulated, whereas its upregulation can restore autophagy and prevent apoptosis. This implies that in normal endothelial cells, *miR-26a* is maintained at a level that supports healthy autophagic flux and prevents apoptosis [17]. lncRNA *GAS5* modulates autophagy in endothelial cells—an essential process for preserving cellular homeostasis and function. ox-LDL-driven endothelial injury and autophagy disruption are key contributors to atherosclerosis. Research data demonstrate concurrent lncRNA *GAS5* elevation and *miR-26a* downregulation in atherosclerotic plasma specimens and oxidized LDL-exposed aortic endothelial cells. By binding to *miR-26a*, lncRNA *GAS5* silencing restores autophagic flux via *miR-26a* elevation, thereby protecting cells from ox-LDL-induced apoptosis and autophagy disruption [17].

The *miR-193-5p*/*serine*/arginine-rich splicing factor 10 (*SRSF10*) pathway: *SRSF10*, which is abundantly expressed in endothelial cells, is an atypical SR protein that functions as a sequence-specific alternative splicing regulator. The knockdown of SRSF10 increases the LC3II/LC3I ratio and decreases p62 levels, thereby enhancing autophagosome formation. Given that *miR-193-5p* promotes autophagy and inhibits SRSF10 (which in turn impairs autophagy), and that its expression is suppressed by lncRNA *GAS5* (a molecule that contributes to atherosclerosis and impairs autophagy), it is suggested that in normal endothelial cells, *miR-193-5p* is maintained at a level that supports healthy endothelial cell autophagy by regulating the expression of SRSF10 [85]. lncRNA *GAS5* overexpression in HAECs inhibits autophagy by targeting *miR-193-5p*, which regulates SRSF10. Therefore, the level of LC3 II relative to LC3 I declines, while P62 shows an increase. Such alterations impair autophagy and foster atherosclerosis [85].

#### 4.3.3. Inflammatory Response in Endothelial Cells

Clinical analyses of coronary artery disease cohorts reveal inverse regulatory relationships between lncRNA *GAS5* and *miR-21* expression profiles. Specifically, lncRNA *GAS5* demonstrates synergistic interactions with TNF-α/*IL-17,* while *miR-21* exhibits inverse associations with multiple pro-inflammatory cytokines (TNF-α, IL-1β, IL-6, IL-17). In parallel, higher lncRNA *GAS5* levels correlate with diminished high-density lipoprotein (HDL) and raised CRP [26]. These associations suggest that lncRNA *GAS5* modulates systemic inflammation partly through *miR-21* repression.

The *miR-29a-3p*/toll-like receptor 4 (*TLR4*) pathway: Myricetin treatment downregulated lncRNA *GAS5* and elevated *miR-29a-3p*, suppressing the TLR4/nuclear transcription factor-κB (NF-κB) pathway, led to diminished ox-LDL-induced HUVECs injury, reduced inflammation, and inhibited endothelial-to-mesenchymal transition (EndMT) [86].

**Figure 7:**
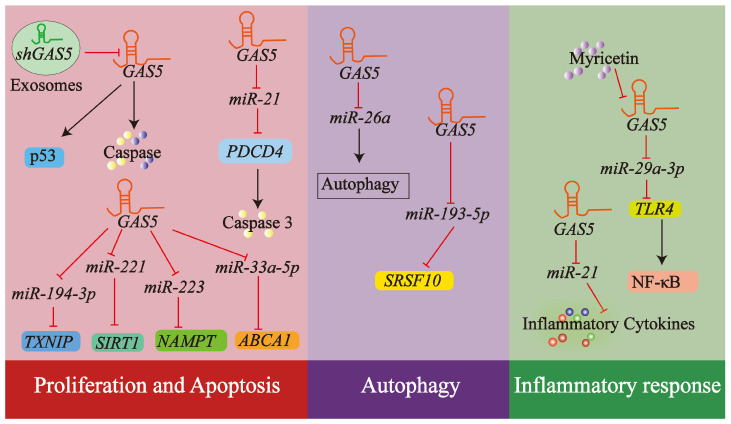
LncRNA *GAS5* signaling networks in ECs dysfunction and atheroprotection/atheroprogression. This diagram highlights the regulatory impact of lncRNA *GAS5* on ECs functions crucial for atherosclerosis, including proliferation, apoptosis, autophagy, and inflammatory responses. *GAS5* exerts its influence via several mechanisms, such as its transfer through exosomes, its role as a ceRNA sequestering multiple miRNAs to derepress target gene expression, and its interplay with other signaling pathways responsive to various stimuli. Arrows and T-bars in the diagram represent promotion/activation and inhibition/suppression. For a comprehensive discussion, see Section 4.3.

In essence, lncRNA *GAS5* is a crucial determinant in maintaining endothelial cell homeostasis by modulating numerous cellular mechanisms, including proliferation, apoptosis, autophagy, and inflammatory responses, all of which are vital for the development and progression of atherosclerosis. Figure 7 offers a visual summary of the key mechanisms through which *GAS5* influences ECs functions relevant to atherosclerosis.

### 4.4. Summary of the Mechanisms of lncRNA GAS5 in Different Cell Types

To more clearly illustrate the complex regulatory network of *GAS5* in key cells of atherosclerosis, the table below summarizes its main interacting molecules, affected signaling pathways, and functional consequences (Table 2).

This table clearly demonstrates that *GAS5* regulates different biological processes through distinct molecular mechanisms in various cells, ultimately exerting complex effects on the development of atherosclerosis. Understanding this cell-specificity and mechanism diversity is crucial for evaluating the overall role of *GAS5* and its feasibility as a therapeutic target.

## 5. Current Therapeutic Applications of lncRNA *GAS5*

### 5.1. Biomarker for Atherosclerosis

lncRNA is considered a potential disease biomarker due to its detectability in tissues and body fluids such as plasma, serum, and exosomes, and its expression levels often change in disease states [87].

One investigation revealed that, within the circulatory compartment of CHD patients, lncRNA *GAS5* is upregulated whereas *miR-21* is downregulated. lncRNA *GAS5* expression demonstrates inverse correlation with HDL-C, yet shows positive associations with CRP concentrations, vascular stenosis severity, and inflammatory mediator levels [26]. Additionally, T2DM can accelerate the progression of carotid atherosclerotic plaques (CAP), predisposing them to instability. RNA sequencing-based analyses indicate differential lncRNA *GAS5* expression between diabetic and non-diabetic cohorts, as well as between unstable and stable CAP groups. Notably, the lncRNA *GAS5*/*miR-30b-3p*/Ras-related protein Rab-37 (RAB37) axis emerges as a functional network governing T2DM and CAP pathogenesis [88].

Future research may need to focus on specific forms of circular *GAS5* (such as exosomal *GAS5*) or be validated in more homogeneous patient populations.

### 5.2. Use of shRNAs or siRNAs

In an in vitro study using siRNA-transfected JS-1 cells, lncRNA *GAS5* overexpression functioned as a ceRNA to reduce *miR-23a* levels. This effect in turn lowered the fibrosis markers α-SMA and collagen I, which reflect hepatic stellate cell (HSC) activation and collagens’ deposition [89]. In a separate experiment, silencing lncRNA *GAS5* raised *miR-194-3p* expression while decreasing TXNIP, thus promoting endothelial cell proliferation and mitigating atherosclerotic plaque formation in an animal model [60].

### 5.3. Small Molecule Inhibitors or Activators

Some findings indicate that lncRNA *GAS5* downregulation hampers glucose uptake and insulin signaling. Following a one-bead-two-compound (OBTC) screen, a small-molecule compound named np-c86 was identified. This agent binds lncRNA *GAS5* with high affinity in adipose-derived stem cells, hinders its interaction with UPF1, and prevents lncRNA *GAS5* degradation, thereby alleviating type 2 diabetes [40]. Moreover, in HT22 cells subjected to siRNA-mediated lncRNA *GAS5* knockdown, TLR8 expression doubled, alongside heightened interferon α (IFN-α), interferon β (IFN-β) transcription, and increased microtubule-associated protein tau (tau) phosphorylation. Treating these cells with np-c86 reversed lncRNA *GAS5* depletion, decreased tau phosphorylation, and constrained the release of inflammatory factors [90]. Myricetin demonstrated attenuation of ox-LDL-mediated endothelial injury, oxidative stress, apoptosis, pro-inflammatory responses, and endothelial transdifferentiation through modulation of the lncRNA *GAS5/miR-29a-3p/TLR4/NF-κB* signaling axis [86].

### 5.4. Gene Editing Approaches

Emerging evidence demonstrates that glucocorticoid exposure suppresses lncRNA *GAS5* expression in human islet tissue and β-cell populations, impairing glucose-regulated insulin output while augmenting apoptotic activity. Crucially, delivery of the lncRNA *GAS5* hormone response element motif (HREM) effectively antagonizes glucocorticoid-mediated lncRNA *GAS5* suppression, restoring insulin production and preserving β-cell viability during dexamethasone challenge [91].

These intervention strategies highlight multiple approaches to modulate *GAS5* activity, ranging from molecular tools (ASO, siRNAs) to pharmacological agents and gene-editing techniques. To systematically compare their mechanisms and therapeutic outcomes, Table 3 summarizes current *GAS5*-targeted interventions, including their molecular targets, biological effects, and applications in atherosclerosis, diabetes, and associated vascular pathologies.

## 6. Challenges and Future Directions

### 6.1. Context-Dependent Expression and Regulation of lncRNA GAS5

As previously discussed, reported expression changes in lncRNA *GAS5* in CHD and diabetic patients require a nuanced interpretation. Distinct studies demonstrate elevated circulating *GAS5* levels in CHD patients [26], whereas serum *GAS5* is significantly reduced in type 2 diabetes mellitus (T2DM) patients, with parallel downregulation observed in adipocytes from T2DM individuals [40].

Potential explanations for these discrepancies may include: Variations in sample types and tissue sources, where *GAS5* expression levels and functional roles differ across biological compartments; heterogeneity in study populations (e.g., ethnicity, comorbidities, disease duration); *GAS5* may exhibit stage-specific or cell-type-selective regulatory behaviors during disease progression; existence of multiple *GAS5* transcript variants, with conflicting results potentially arising from measurement of total *GAS5* versus specific splice isoforms across studies.

### 6.2. Current Challenges

The mechanisms of *GAS5* are complex and diverse, involving multiple interacting molecules and signaling pathways, and exhibit significant cell-type and environment dependency. The interactions and hierarchical relationships between different mechanisms are not yet fully understood [59,92].

*GAS5* has different splicing isoforms (such as *GAS5a*, *GAS5b*), but their specific functions and regulatory mechanisms in atherosclerosis have not been sufficiently studied [92].

The value of circulating *GAS5* as a biomarker has not been consistently validated and requires larger, more rigorously designed clinical studies for verification. Additionally, due to the high cell-specificity of *GAS5* function, developing safe and effective *GAS5*-targeted therapeutic strategies faces challenges related to delivery and specificity [59,93].

### 6.3. Future Directions

To gain a deeper understanding of *GAS5*’s role in atherosclerosis and to facilitate its clinical translation, future research should focus on the following areas:

(1) Elucidate the precise molecular interaction network of *GAS5* in various relevant vascular cells (ECs, VSMCs, macrophages, and other immune cells), identifying additional miRNA targets and protein partners, as well as the downstream signaling pathways they regulate; differentiate the expression patterns and functional differences among various *GAS5* splicing isoforms, clarifying their specific roles throughout the various developmental phases of atherosclerosis and within diverse cellular environments.
(2) Conduct large-scale, multicenter, prospective clinical cohort studies to rigorously evaluate the diagnostic and prognostic value of circulating *GAS5* (including total levels and exosomal levels) across different subtypes and stages of cardiovascular disease and explore its potential for use in combination with other biomarkers.
(3) Employ various methods, such as those described in Section 5 for regulating *GAS5* in other diseases, to achieve precise control of *GAS5* expression or function in specific cells; additionally, investigate the feasibility of targeting key downstream effectors of *GAS5*.
(4) Utilize research models closer to human pathophysiological states, such as humanized mouse models and vascular organoids, along with single-cell multi-omics analysis of human atherosclerotic plaques, to better bridge basic research and clinical practice.
(5) Investigate the interaction (crosstalk) and feedback loops between *GAS5* and additional non-coding RNAs, like other lncRNAs and circular RNAs (circRNAs), along with the epigenetic regulatory network, to construct a more comprehensive map of the atherosclerosis regulatory network.

## 7. Conclusions

In summary, lncRNA *GAS5* exerts multifaceted and critical functions in atherosclerosis onset and progression. lncRNA *GAS5* modulates miRNA networks and signaling axes to coordinate core regulatory circuits in heterogeneous cellular populations driving atherogenic mechanisms.

The spectrum of lncRNA *GAS5*’s molecular activities—which includes acting as a ceRNA to sequester various miRNAs—underscores its influential role in gene expression regulation. Because aberrant lncRNA *GAS5* expression correlates with atherosclerosis pathogenesis, it represents a noteworthy candidate biomarker for both diagnostic and prognostic purposes.

From a therapeutic viewpoint, targeting lncRNA *GAS5* holds considerable potential. Strategies involving antisense oligonucleotides, small-molecule inhibitors or activators, and gene editing technologies have already shown promise in modulating lncRNA *GAS5* expression and functionality. These interventions could lead to the development of novel therapeutics aimed at preventing or reversing atherosclerosis progression.

Nevertheless, challenges persist in fully elucidating the upstream factors modulating lncRNA *GAS5*, defining its broader interactions within cellular networks, and translating these discoveries into clinical practice. Future investigations should employ state-of-the-art techniques—such as single-cell sequencing, CRISPR-Cas9 genome editing, and RNA-based therapeutics—to dissect lncRNA *GAS5*’s in vivo modes of action and pave the way for targeted therapies.

Ultimately, achieving a comprehensive understanding of lncRNA *GAS5* in atherosclerosis holds substantial promise for enhancing patient outcomes. Harnessing the regulatory capacity of lncRNA *GAS5* can potentially foster improved diagnostic methodologies and therapeutic strategies for atherosclerosis. Ongoing exploration of lncRNA *GAS5*’s molecular underpinnings and therapeutic applications remains crucial for advancing cardiovascular medicine and mitigating the global impact of atherosclerotic disease.

## Figures and Tables

**Table 1:** Summary of the targets and related functions of lncRNA *GAS5* in different diseases.

Disease Type	Interacting Molecules/Signaling Pathway	Related Functions	Ref
Cancer	*miR-21*	Tumor-Inhibiting	[42]
	*miR-21*/*PTEN*	Tumor-Inhibiting	[50]
	*miR-18a*	Tumor-Inhibiting	[51]
Cardiovascular disease	β-catenin signaling	Vascular Remodeling	[48]
	sema3a	Cardiomyocyte Apoptosis	[52]
	YBX1, *miR-21*	VSMCs Apoptosis	[45]
Endocrine and metabolic diseases	UPF1	Glucose Uptake	[40]
Nervous system diseases	PRC2/IRF4	Microglial Polarization	[53]
Bone and development	*miR-144*/*mTOR*	Autophagy of Chondrocytes	[54]
	*miR-291-a*, cMyc	Self-Renewal and Pluripotency of Stem Cells	[55]

**Table 2:** Summary of cell-specific mechanisms of lncRNA *GAS5* in atherosclerosis.

Cell Type	Species	Key Interacting Molecules/Signaling Pathway	Biological Process Affected	Ref
VSMCs	Rat	p53-p300, p21, NOXA	Cell cycle arrest (G1), Apoptosis, Proliferation	[67]
	Human	*miR-21*/*PDCD4*	Proliferation, Migration	[68]
	Mouse	*miR-145-5p*	Proliferation, Apoptosis	[30]
	Rat	ATPase, L31	Proliferation, Phenotypic switching	[29]
	Human/Rat	*miR-21*, Akt/ERK pathway	Migration	[68,69]
	Rat	*miR-23b-3p*/*KCNK3*	Proliferation, Migration, Phenotypic switching	[70]
	Human/Mouse	*miR-665*/*SDC1*	Anti-senescence, Phenotypic switching	[71,72,73]
Macrophages	Human	*miR-221*/*MCP*-1	Inflammatory response	[74]
	Human	*miR-135a*	Inflammatory response, Oxidative stress	[56,75]
	Mouse	ANXA2	Inflammatory response	[64]
	Human	*miR-221*, MMP-2, MMP-9	MMP biosynthesis, ECM degradation	[74]
	Human	*miR-135a*	Lipid metabolism	[56]
	Human	EZH2, ABCA1, ABCG1	Lipid accumulation, Cholesterol efflux	[32]
	Human	*miR-128-3p*/*FBLN2*	Proliferation, Apoptosis	[77]
	Mouse	ANXA2	Apoptosis	[64]
	Mouse	*miR-145-5p*	Foam cell formation, Lipid uptake, Plasma lipids	[30]
ECs	Human	p53, Caspases	Apoptosis	[57]
	Human	*miR-21*/*PDCD4*, Caspase	Apoptosis, Proliferation	[66]
	Rat	*miR-194-3p*/*TXNIP*	Proliferation, Apoptosis	[60]
	Human	*miR-223*/*NAMPT*/*PI3K*/*AKT*	Proliferation, Senescence	[83]
	Human	*miR-221*/*Sirt1*	Proliferation, Migration	[81]
	Rat	*miR-33a-5p*/*ABCA1*	Proliferation, Apoptosis	[84]
	Human	*miR-26a*	Autophagic flux, Apoptosis	[17]
	Human	*miR-193-5p*/*SRSF10*	Autophagy	[85]
	Human	*miR-23a-3p*/*TLR4*	Endothelial growth, Inflammation, EndMT	[86]

This table summarizes key cell-specific mechanisms of lncRNA *GAS5* in atherosclerosis as detailed in Section 4. Abbreviations: ECs, Endothelial Cells; miR, microRNA; VSMCs, Vascular Smooth Muscle Cells.

**Table 3:** lncRNA *GAS5*-targeted therapeutic strategies.

Methods	Effect	Disease Domain	Ref
siRNA	*GAS5* downregulation and liver fibrosis indicators were upregulated	Hepatic fibrosis	[89]
shRNA	*GAS5* downregulation, protects endothelial cell function, and reduces atherosclerosis	Coronary atherosclerosis	[60]
np-c86	*GAS5* upregulation and promotes insulin receptor activity and glucose absorption	Diabetes	[40]
np-c86	*GAS5* upregulation, improves neuronal insulin signaling, and reduces neuroinflammation	Neurodegenerative diseases	[90]
Myricetin	*GAS5* downregulation, inflammatory response and EndMT reduction	Atherosclerosis	[86]
ASO/HREM	*GAS5* was downregulated/upregulated, and β cell activity was enhanced when it was upregulated	Diabetes	[91]

## Data Availability

No data were used for this review paper.
